# Lipid Regulatory Proteins as Potential Therapeutic Targets for Ovarian Cancer in Obese Women

**DOI:** 10.3390/cancers12113469

**Published:** 2020-11-21

**Authors:** Jing Yang, M. Sharon Stack

**Affiliations:** 1 Department of Chemistry and Biochemistry, University of Notre Dame, South Bend, IN 46617, USA; jyang@nd.edu; 2 Harper Cancer Research Institute, University of Notre Dame, South Bend, IN 46617, USA

**Keywords:** obesity, ovarian cancer, SREBP1, lipogenesis, metastasis

## Abstract

**Simple Summary:**

Obesity is a recognized epidemic worldwide. Ovarian cancer (OvCa), the most lethal gynecologic malignancy, is characterized by widely disseminated metastatic implants in the peritoneal cavity. Metastatic lesions infiltrate the omentum, as well as perigonadal adipose and other visceral adipose depots. Meta-analyses support a link between obesity and OvCa progression, metastasis and survival. Recent studies demonstrated a crucial role of lipid regulatory proteins in OvCa growth and metastasis. The aim of this review is to summarize current understanding of the relationship between lipid regulatory proteins, obesity and OvCa progression, as well as the potential therapeutic targets for antitumor therapy in obese OvCa patients, with an emphasis on the role of sterol regulatory element binding protein 1 (SREBP1).

**Abstract:**

Obesity has become a recognized global epidemic that is associated with numerous comorbidities including type II diabetes, cardiovascular disease, hypertension, and cancer incidence and progression. Ovarian cancer (OvCa) has a unique mechanism of intra-peritoneal metastasis, already present in 80% of women at the time of diagnosis, making it the fifth leading cause of death from gynecological malignancy. Meta-analyses showed that obesity increases the risk of OvCa progression, leads to enhanced overall and organ-specific tumor burden, and adversely effects survival of women with OvCa. Recent data discovered that tumors grown in mice fed on a western diet (40% fat) have elevated lipid levels and a highly increased expression level of sterol regulatory element binding protein 1 (SREBP1). SREBP1 is a master transcription factor that regulates de novo lipogenesis and lipid homeostasis, and induces lipogenic reprogramming of tumor cells. Elevated SREBP1 levels are linked to cancer cell proliferation and metastasis. This review will summarize recent findings to provide a current understanding of lipid regulatory proteins in the ovarian tumor microenvironment with emphasis on SREBP1 expression in the obese host, the role of SREBP1 in cancer progression and metastasis, and potential therapeutic targeting of SREBPs and SREBP-pathway genes in treating cancers, particularly in the context of host obesity.

## 1. Introduction

The prevalence of overweight and obesity has increased substantially and become a major health issue worldwide [1]. Body mass index (BMI) is a simple index of weight-for-height that is commonly used in clinical medicine and population health. Recent epidemiological studies show that the age-adjusted prevalence of combined overweight and obesity (BMI ≥ 25 kg/m^2^) was 68.8% overall, 73.9% among men and 63.7% among women [2,3]. In 2017–2018, the age-adjusted prevalence of obesity (BMI ≥ 30 kg/m^2^) was 42.4% among U.S. adults aged 20 and over [4]. Obesity greatly increases the risk of common chronic diseases and metabolic morbidity such as type II diabetes, cardiovascular disease, stroke, hypertension, dyslipidemia and osteoarthritis [5]. Moreover, strong epidemiologic evidence shows that obesity and excess accumulation of adipose tissue is associated with increased risk of numerous solid and hematologic cancer incidence and progression [6,7], and a high BMI has been considered as a negative prognostic factor for oncological outcomes, cancer recurrence and mortality [8].

Ovarian cancer (OvCa) is the leading cause of death from gynecologic malignancy [9,10]. Survival of women diagnosed with OvCa has not changed appreciably in over 30 years and most women will die from painful complications that arise as a result of widely disseminated intra-peritoneal (i.p.) metastasis [11,12,13,14]. Compared with other cancers, OvCa has a unique molecular and cellular tumor microenvironment (TME) to promote tumorigenesis and metastasis, evade immune surveillance and mediate therapy resistance [15]. Tumors are genetically highly heterogeneous and exhibit diffuse patterns of metastasis to the adipose-rich omental fat pad and multiple sites within the peritoneum [13,14,15,16,17]. Resident host cells, including activated mesothelial cells which line the peritoneal cavity [18], the omental adipocytes and localized immune aggregates (milky spots) constitute the preferred sites of OvCa metastasis [15,19,20,21,22]. Meta-analyses showed that obesity increases the risk of OvCa incidence and progression, and adversely effects the survival of women with OvCa, implicating a link between host obesity and metastatic success [23,24,25,26,27].

Recent murine in vivo studies demonstrated that genetic and diet-induced obesity leads to enhanced OvCa tumor burden, increased intracellular lipid content, and elevated expression of sterol regulatory element binding protein 1 (SREBP1) [28]. SREBP1 is a master regulator of de novo lipogenesis and lipid homeostasis [29,30,31,32]. SREBP1 expression was significantly higher in the metastatic OvCa [33,34,35], and knockdown of SREBP1 inhibited ovarian tumor growth in vivo [34]. Enhanced expression of SREBP1 induces lipogenic reprogramming of tumor cells and promotes cancer cell proliferation and metastasis [36,37,38].

In this review, we discuss the current understanding of ovarian cancer metastasis mechanisms, summarize recent findings that obesity contributes to cancer progression with a focus on the role of lipid regulatory protein SREBP1 in regulating cancer progression and metastasis, and discuss potential therapeutic targets in obese cancer patients.

## 2. Mechanism of Ovarian Cancer Metastasis

Ovarian cancer is the most lethal gynecological malignancy in the United States [9,10]. The majority (90%) of OvCa are of epithelial origin and are further classified into five histopathological subtypes: high-grade serous carcinoma, endometrioid, clear cell, mucinous, and low-grade serous carcinoma [39]. Among them, high-grade serous ovarian cancer (HGSOC) is the most common malignant histotype accounting for up to 70% of all OvCa cases [12]. When diagnosed at an early stage prior to metastatic dissemination, the overall 5-year survival rate is 92%. However, due to the lack of effective early screening strategies and nonspecific symptoms at early disease stages, most ovarian cancer patients (75%) are diagnosed at late stages III−IV when tumors have metastasized throughout the pelvic and peritoneal cavity at which point the 5-year survival substantially drops to 29% [11,12,13]. 

**Figure 1:**
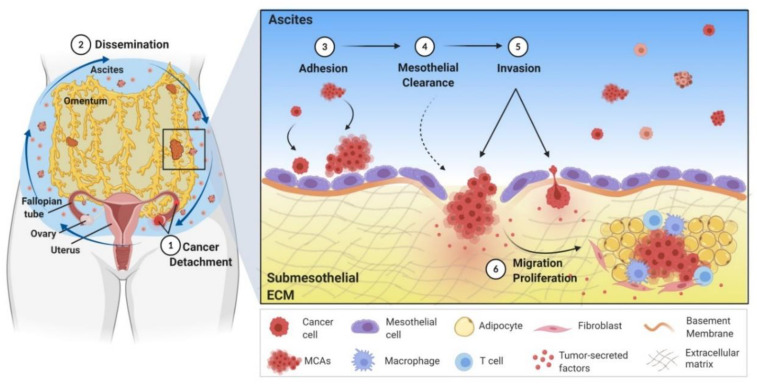
Transcoelomic dissemination model of ovarian cancer metastasis. (1) Ovarian cancer cells are exfoliated from the primary ovarian surface or fallopian tubes into the peritoneal cavity. (2) These detached single cells and multicellular aggregates (MCAs or spheroids) float in the peritoneal fluid or ascites and disseminate throughout the peritoneal cavity, as depicted by the blue arrows. Ovarian cancer cells and MCAs adhere to and implant on the peritoneum and peritoneal organs such as the omentum (3), where they clear the mesothelial lining (4), invade into the submesothelial extracellular matrix (ECM) (5), migrate and proliferate to form multiple distal metastatic tumors (6).

The high mortality rate among women diagnosed with advanced stages of OvCa is directly attributable to widely disseminated intra-peritoneal (i.p.) metastasis [14]. Ovarian tumors originate in the epithelial cells of both fallopian tubes (FTE) and ovarian surface (OSE) [40] and subsequently colonize the ovaries and/or peritoneum (Figure 1) [13,41,42,43]. These tumors are highly heterogeneous with multiple genetic and epigenetic abnormalities [44]. Approximate 20% HGSOC patients have hereditary germline mutations of *BRCA1* or *BRCA2* [44,45,46]. *BRCA1/2*-mutated OvCa populations are sensitive to poly(ADP-ribose) polymerase (PARP) inhibitors and to date four PARP inhibitors are approved for use in maintenance therapy for recurrent OvCa patients [47,48]. In addition, *TP53* gene mutations occur in almost 100% of HGSOCs [44,49,50,51,52]. 

The primary mechanism of OvCa metastasis is via shedding or extension into the peritoneal cavity via a direct transcoelomic dissemination route (Figure 1) [13,14,53]. Upon detaching from the primary ovarian surface or fallopian tubes, OvCa cells survive as anchorage-independent single cells and multicellular aggregates (MCAs or spheroids) in the peritoneal cavity. Large volumes of peritoneal fluid (ascites) are commonly found in the majority of OvCa patients (89%) with advanced disease (stages III and IV). Malignant ascites provides an additional vehicle for i.p. OvCa cell dissemination as well as a microenvironment to support tumor cell survival and influence cellular behavior [54]. Disseminating OvCa cells and MCAs float in the peritoneal ascites fluid, subsequently adhere to the mesothelial cells (MC) of the peritoneal membrane, induce MC retraction to facilitate anchoring in the sub-mesothelial collagen-rich extracellular matrix (ECM), and then proliferate to form secondary lesions (Figure 1) [13,14,55]. 

Hematogenous metastasis of OvCa cells has recently been reported [56,57]. In these studies, OvCa cells at the primary tumor site invade through the basement membrane of blood vessels and enter the circulation via intravasation. The circulating OvCa cells can then leave the circulation via extravasation, whereupon they preferentially implant and grow in the omentum, ovary and subsequently spread to other peritoneal surfaces [14,56,57]. Despite the differences between these two mechanisms of ovarian cancer metastasis, OvCa cells possess a distinct tumor tropism predominately for the adipose-rich omentum and peritoneal surfaces to establish secondary metastatic tumor nodules. [13,14,15,16,17]. 

## 3. The Link between Obesity and Ovarian Cancer

### 3.1. Obesity-Associated Tumor Inflammatory Microenvironment Contributes to Ovarian Cancer Progression and Metastasis

Accumulating evidence supports the significance of obesity to ovarian tumor progression and metastasis [6,58,59] with the unique tumor microenvironment (TME) playing a critical role linking obesity to cancer. OvCa has a unique molecular and cellular TME to promote tumorigenesis and metastasis, evade immune surveillance and mediate therapy resistance [60,61,62,63,64,65,66]. The characteristic features of the OvCa TME are: (1) resident host cells, in particular huge numbers of activated mesothelial cells which line the peritoneal cavity [18] and omental adipocytes that constitute the preferred site of OvCa metastasis [15]; (2) an increased volume of peritoneal fluid in the form of ascites, which generates a rich and intricate microenvironment composed of a network of heterogeneous cell types and tumor-promoting soluble factors, including detached tumor cells [67], infiltrating immune cells such as T cells [68] and tumor-associated macrophages (TAMs) [69,70], inflammatory cytokines and growth factors [71], exosomes [72] and other host cells. Ascites fluid builds up by malignancy-associated effusion into the peritoneal cavity to facilitate the transcoelomic dissemination of tumor cells to other pelvic and peritoneal organs [73]. 

During metastatic dissemination, OvCa cells preferentially home to adipose tissue and adipocytes located in the visceral omentum, a large fat pad which is positioned in front of the small bowel and extends to the pelvis [74]. When OvCa cells are cocultured with human omental adipocytes or with their conditioned media, increased cancer cell proliferation, migration, invasion and tumor growth were observed [19,75]. 

Obesity is a recognized cause of chronic inflammation, both systemically and locally at the tissue level. Obese adipose tissue is infiltrated by multiple immune cells including macrophages and lymphocytes. This resembles chronically injured tissue in a persistent inflammatory state characterized by secretion of adipokines and cytokines fostering tumor growth, such as interleukins (IL) IL-8, IL-6, monocyte chemoattractant protein-1 (MCP-1), tissue inhibitor of metalloproteinases-1 (TIMP-1), adiponectin, tumor necrosis factor (TNF)-α, as well as production of vascular endothelial growth factor (VEGF), prostaglandins and leukotrienes by activated macrophages [58,76,77,78]. These proinflammatory mediators attract OvCa cells to the omentum. For example, adipocyte-secreted IL-8 binds to CXCR1 whose expression is upregulated in OvCa cells to induce p38 mitogen-activated protein kinase and STAT3 phosphorylation, hence promoting the initiation of OvCa metastasis [19].

### 3.2. Obese Adipose Tissue Promotes Ovarian Cancer Progression by Altered Exogenous Lipid Transport and Uptake

Once the OvCa cells interact with omental adipocytes, the adipocytes are induced to initiate hormone-sensitive lipase (HSL)-mediated lipolysis of triglycerides to release fatty acids (FAs). The uptake and scavenging of extracellular FAs by the surrounding metastatic OvCa cells provides the cancer cells with a compensatory mechanism to sustain increased lipid demands under metabolic stress. The exogenous uptake and transport of FAs is facilitated by specialized transporters and FA binding proteins. For example, CD36 (also known as FA translocase (FAT)) is a transmembrane glycoprotein that mediates exogenous uptake of long chain FAs and cholesterol, as well as transduces intracellular signaling that regulates the metabolic targeting of FAs [75,79]. Upregulation of CD36 with altered cellular energy homeostasis has been found in human metastatic ovarian tumors. Silencing of CD36 by shRNA knockdown in OvCa cells reduced the uptake of microenvironment-derived FA and cholesterol, decreased lipid droplet accumulation and attenuated intracellular reactive oxygen species (ROS) content. CD36 inhibition also impairs OvCa cell adhesion, migration, invasion, and clonogenic capacity in vitro, and reduces tumor growth in vivo [75]. 

Similarly, enhanced expression of FABP4 (fatty acid-binding protein 4) was found both upon in vitro OvCa-adipocyte cell coculture and in omental metastatic ovarian tumors [19,28,80,81]. FABP4, a lipid chaperone protein primarily expressed in adipocytes and macrophages, coordinates cellular lipid responses by binding to and redistributing intracellular FA. Hypoxia decreases microRNA miR-409-3p which binds to the 3′UTR of FABP4 and negatively regulates FABP4 in OvCa [81]. CRISPR-mediated knockdown of FABP4 in OvCa reduced lipid droplet accumulation, decreased adipocyte-induced but not constitutive β-oxidation, and blocked adipocyte-induced ROS generation. Inhibition of FABP4 reduced metastatic tumor burden in vivo [80,81], and also increased the sensitivity of OvCa cells toward carboplatin when treated with a small-molecule inhibitor of FABP4 (BMS309403) [80].

Once inside the cancer cell cytosol, FAs are used for lipid synthesis in the membrane or as transcriptional regulators in the nucleus, while excess FAs are sequestered and stored in the cytoplasmic organelles lipid droplets (LDs) [82]. LDs are accumulated in cancer cells to maintain lipid homoeostasis and to provide an energy source during metabolic stress [83]. This is achieved through β-oxidation of LD-stored lipids in mitochondria, producing acetyl-CoA which enters the tricarboxylic acid (TCA) cycle to generate NADH and FADH_2_, ultimately leading to the synthesis of ATP [84]. The transfer of FAs from microenvironmental adipocytes and uptake of FAs in metastatic ovarian cancer cells induce AMP-activated protein kinase (AMPK), an energy sensor kinase that regulates energy production by activating β-oxidation. AMPK phosphorylates acetyl-CoA carboxylase (ACC) to inhibit enzyme activity, leading to reduced intracellular malonyl-CoA and increased carnitine palmitoyltransferase 1 (CPT1) which regulates the mitochondrial import of fatty acids for β-oxidation [19,85].

### 3.3. Obesity Promotes Ovarian Cancer Progression by Altered de novo Lipogenesis

In normal tissue, de novo lipogenesis (DNL) is a regulated metabolic process that converts excess carbohydrates into FAs mainly in hepatocytes and adipocytes. Extensive studies have demonstrated that cancer cells obtain lipid not only from exogenous uptake but also from increasing DNL synthesis [86,87,88]. 

Cancer cells exploit carbohydrate sources including glucose, glutamine and acetate to synthesize citrate for DNL. Acetyl-CoA derived from citrate or acetate is the main cellular substrate for FA synthesis. Under metabolic stress such as hypoxia or lipid depletion, cancer cells upregulate acetyl-CoA synthetase 2 (ACSS2) to generate acetyl-CoA from acetate [89], or increase ATP-citrate lyase (ACLY) to convert citrate into acetyl-CoA [90]. Saturated 16-carbon FA palmitate (FA16:0) is generated from citrate through the sequential enzymatic activities of ACLY [90], acetyl-CoA carboxylase (ACC) [91] and fatty acid synthase (FASN) [92]. Palmitate is subsequently desaturated by stearoyl-CoA desaturase (SCD) to produce monounsaturated fatty acids (MUFAs) [93,94], or by fatty acid desaturase 2 (FADS2) to generate sapienate [95]. MUFAs are then elongated by FA elongases to form a variety of polyunsaturated FAs (PUFAs). DNL pathways including lipid synthesis followed by downstream desaturation and elongation are upregulated in cancers to satisfy the demands of enhanced membrane biogenesis, increase adaptability to cytotoxic stress in TME, and act as a compensatory biosynthetic pathway to generate a variety of lipid species with distinct functions.

Using three distinct murine genetic and diet-induced obesity models, we recently reported that host obesity leads to significantly enhanced overall and organ-specific metastatic OvCa tumor burdens, increased intra-cellular lipid content, and elevated expression of sterol regulatory element binding protein 1 (SREBP1) [28]. SREBP1 was found highly expressed in the metastatic OvCa [33,34,35], and knockdown SREBP1 gene expression inhibited ovarian tumor growth in vivo [34]. Among SREBP1-regulated FA synthesis genes, *ACLY, ACC, FASN, and SCD1* have been reported to be highly expressed in cancers [33,92,96,97,98,99,100]. These data demonstrated that in addition to elevated fatty acid transport, SREBP1-directed upregulation of de novo fatty acid synthesis also contributes to enhanced OvCa growth and metastasis in the obese host microenvironment.

Together these findings demonstrate metabolic plasticity and bioenergetic adaptation of OvCa cells in the adipocyte-rich microenvironment. Adipocytes act as an energy source for the cancer cells, stimulate mitochondrial metabolism in cancer cells and support tumor growth, all of which are essential for driving the progression and metastasis of ovarian cancer. 

## 4. Sterol Regulatory Element Binding Protein (SREBP)-Regulated *de novo* Lipogenesis in Cancer

### 4.1. SREBP Pathway

**Figure 2:**
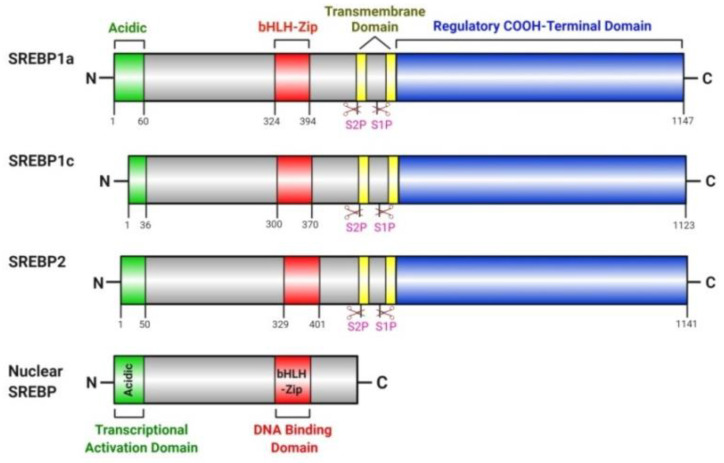
Domain structure of human sterol regulatory element binding proteins (SREBPs). Each SREBP precursor (SREBP1a, SREBP1c and SREBP2) shares a similar hairpin-like structure containing about 1150 amino acids (~125 KDa) and is organized into three domains: (1) an NH2-terminal cytoplasmic domain of about 480 amino acids (~68 KDa) that consist of an acidic transcriptional activation domain and a basic helix-loop-helix-leucine zipper (bHLH-Zip) DNA binding domain; (2) a central hydrophobic region containing two transmembrane segments that project into the ER lumen; (3) and a COOH-terminal cytoplasmic regulatory domain of about 590 amino acids. The NH2-terminal nuclear forms of mature SREBPs (nSREBPs) are generated by the two sequential proteolytic cleavages within the central region of the precursors upon sterol depletion.

SREBPs are the key transcription factors that regulate de novo lipogenesis and lipid metabolism by controlling the expression of enzymes required for cholesterol, fatty acids, triglycerides and phospholipid biosynthesis under both physiological and pathological conditions [29,30,31]. Two SREBP genes, SREBF-1 and SREBF-2, express three SREBP isoforms in mammals. SREBP1 isoforms -1a and -1c are generated through the use of alternative promoters of SREBF-1. SREBP1c has a shorter N-terminal transactivation domain and thus weaker transcriptional activity (Figure 2). SREBP1c governs fatty acid and triglyceride synthesis mainly in lipogenic organs such as the liver, whereas SREBP1a primarily regulates global lipid synthesis in proliferating cells and other organs. SREBP2 is derived from the SREBF2 gene and ubiquitously regulates cholesterol synthesis in tissues [101,102]. 

SREBPs are synthesized in the endoplasmic reticulum (ER) and reside as inactive precursors in a hairpin orientation such that the N- and C-termini project into the cytosol. These termini are separated by two transmembrane segments that surround a short luminal loop. The N-terminal domain of SREBPs (nSREBP) is a transcription factor of the basic helix-loop-helix-leucine zipper (bHLH-LZ) family. Proteolytic cleavage frees nSREBP to move to the nucleus and bind to specific sterol regulatory elements (SREs) in the promoter region of the genes involved in lipid biosynthesis. The C-terminal domain of SREBPs performs a regulatory function and forms a complex with the WD repeats in the C-terminal domain of SREBP cleavage-activating protein (SCAP) (Figure 2). 

**Figure 3:**
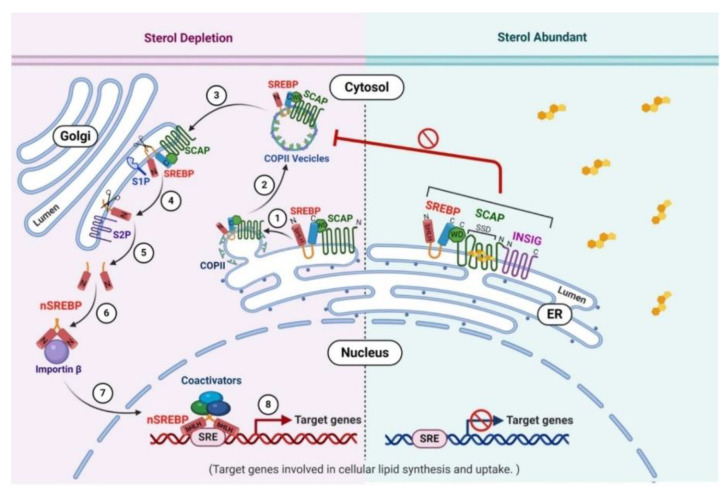
Schematic illustration of sterol-regulated proteolytic activation of SREBPs. **Right**: Under conditions when intracellular sterol levels are elevated, it directly binds to the sterol-sensing domain (SSD) of SCAP, causing a conformational change in SCAP which triggers SCAP bind to another ER-resident membrane protein INSIG. The association of SREBP-SCAP-INSIG ternary complex results in retention of SREBP precursors in the ER and prevents the movement of SREBP-SCAP complex to the Golgi, thus inhibiting the proteolytic maturation of SREBP in the Golgi. **Left**: In the absence of sterol, INSIG is no longer associated with SREBP-SCAP in the ER (1), and SREBP-SCAP complex is loaded into COPII vesicles which bud out of the ER membrane (2) and subsequently fuse with the Golgi apparatus (3). In the Golgi, Site-1 Protease (S1P) carries out the first proteolytic cleavage (scissor) in the intra-luminal loop of SREBPs between the two membrane-spanning sequences (4). Once the two halves of the SREBP are separated, a Golgi intramembrane metalloprotease Site-2 Protease (S2P) accomplishes the second cleavage (scissor) at a site located within the membrane-spanning region in the NH2-terminal domain of SREBP, releasing the bHLH-Zip domain which represents the mature SREBP (nSREBP) (5). Two nSREBP fragments dimerize and interact with importin β (6), and translocate to the nucleus (7) where nSREBP bind to sterol regulatory element (SRE) sequences as a dimer to gene promoter/enhancer regions and activate target genes that control synthesis and uptake of cholesterol, fatty acids, triglycerides, and phospholipids (8). SREBP, sterol regulatory element-binding protein; SCAP, SREBP cleavage-activating protein; SSD, sterol-sensing domains; WD: Tryptophan-Aspartate repeat motif; INSIG, insulin-induced gene; COPII, coat protein II; ER, endoplasmic reticulum; S1P, site-1 protease; S2P, site-2 protease; SRE, sterol regulatory element.

Cells properly use the SREBP pathway regulated intramembrane proteolysis to maintain their intracellular lipid homeostasis through regulatory feedback machinery. When intracellular cholesterol levels are increased (Figure 3, right), a conformational change in SCAP occurs through cholesterol directly binding to the sterol-sensing domain of SCAP, and triggers SCAP binding to another ER-resident membrane protein insulin-induced gene (INSIG). The association of SREBP-SCAP-INSIG complex results in retention of SREBP precursors in the ER and prohibits the ER-to-Golgi transport of SREBP-SCAP. This inhibits the proteolytic maturation of nSREBP in the Golgi and blocks SREBP from acting as a transcription factor which ultimately results in decreased expression of lipid biosynthesis genes.

When cholesterol levels are depleted (Figure 3, left), the sterol-sensing domain (SSD) of SCAP undergoes a conformational change that exposes a hexapeptide sorting signal (MELADL), which allows for its interaction with COPII vesicles coated proteins. SCAP, escorting SREBP along with it, is incorporated into the COPII vesicles which bud from the ER membrane and transport to the Golgi. SREBP then undergoes two sequential proteolytic cleavages in the Golgi, where site-1 protease (S1P) catalyzes the initial cleavage within the short luminal loop followed shortly thereafter by site-2 protease (S2P) catalyzed cleavage within the first transmembrane segment of SREBP [36,103,104,105,106]. The released nSREBP is transported into the nucleus as a dimer and activates transcription by binding to the SRE sequence in the promoters of target genes involved in lipogenesis, such as low-density lipoprotein receptor (LDLR), ACLY, ACC, FASN, and SCD1 [32,107,108,109]. Synthesis of sterols then, in turn, inhibits the cleavage of SREBPs through a negative feedback loop. Mature nSREBPs are unstable and rapidly degraded by ubiquitination in the proteasome pathway [110,111].

### 4.2. Regulation of de novo Lipogenesis by SREBP in Cancer

Lipidomic reprogramming is a metabolic hallmark of cancer [88,112,113,114]. Accumulating evidence has demonstrated that increased lipid synthesis is strongly correlated with cancer cell proliferation, invasion and metastasis [37,112]. To satisfy the demands of increased membrane biogenesis and constant replication, cancer cells must sustain high levels of lipid through both increased de novo biosynthesis and exogenous uptake [86,115]. Besides their primary role as structural components of the cell membrane, lipids also function as second messengers for oncogenic signaling as well as fuel sources for energy production and storage. Lipids such as cholesterol, fatty acids, oxysterols, and triglycerides that are generated through activation of the SREBP pathway are highly expressed in cancers and stimulate tumorigenesis [116,117,118]. For example, the cholesterol metabolite 27-hydroxycholesterol that is produced within tumors acts as an agonist for the estrogen receptor and the liver X receptor and promotes estrogen receptor-positive breast cancer growth and metastasis [118,119]. In clear cell renal cell carcinoma, increased levels of unsaturated fatty acids stimulate cell proliferation by inhibiting β-catenin degradation [120].

**Figure 4:**
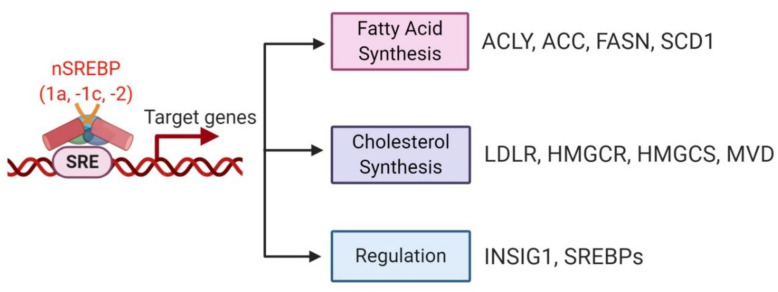
Downstream target genes of SREBPs. The homo- and heterodimerized nSREBPs (-1a, -1c and -2) enter the nucleus, in combination with other coactivators, bind to sterol regulatory elements (SREs) in the promoters/enhancers and activate transcription of target genes. SREBP-1a, -1c specifically activate genes involved in fatty acid synthesis, such as *ACLY, ACC, FASN* and *SCD1.* SREBP2 preferentially targets genes involved in cholesterol biosynthesis, such as *LDLR, HMGCR, HMGCS*, and *MVD*. INSIGs are negative regulators of SREBP proteolysis, while the *INSIG1* gene itself is a target of the nSREBPs. Thus *INSIG1* is also regulated by SREBPs. As a target gene of itself, *SREBPs* mRNA can be induced and regulated by nSREBPs as well. ACLY, ATP citrate lyase; ACC, acetyl-CoA carboxylase; FASN, fatty acid synthase; SCD1, stearoyl-CoA desaturase 1; HMGCS, HMG-CoA synthase; HMGCR, HMG-CoA reductase; LDLR, low density lipoprotein receptor; MVD, diphosphomevalonate decarboxylase; INSIG1, insulin-induced gene 1.

SREBPs [28,38] regulate DNL through activation of the key downstream lipogenic enzymes (Figure 4). SREBPs upregulate the expression of ACLY [90,97,121], ACC [98,122], FASN [92,96,98,123,124] and SCD1 [99] to promote FA synthesis, as well as LDLR, HMG-CoA reductase (HMGCR), HMG-CoA synthase (HMGCS) and diphosphomevalonate decarboxylase (MVD) to enhance cholesterol uptake and synthesis. These essential SREBP pathway factors are aberrantly activated and highly expressed in various types of cancers, including breast cancer, prostate cancer, hepatocellular carcinoma, glioblastoma, pancreatic cancer, colorectal carcinoma, and ovarian cancer [28,34,38,125,126,127,128,129,130,131,132,133,134,135,136]. Inhibition of these regulators either genetically or pharmacologically showed significant reduction of cancer cell growth in vitro and in vivo [90,97,122,123,124,137,138,139].

### 4.3. Regulation of SREBP by Oncogenic and Tumor Suppressor Signaling Pathways

Besides sterol, cancer cells develop multiple pro-tumorigenic signaling molecules and redundant molecular pathways to control SREBP expression and maintain the stability of mature nSREBP, thereby guaranteeing production of sufficient lipids for rapid cell growth and proliferation [140]. For example the PI3K/Akt/mTORC oncogenic signaling pathway, frequently activated in various cancers including ovarian cancer, plays a pivotal role in processing of SREBPs and regulating DNL [141,142,143,144]. Activation of receptor tyrosine kinases (RTKs) through growth factor binding recruits phosphatidylinositol-3-kinase (PI3K) to the plasma membrane, where it phosphorylates inositol-containing membrane lipids like phosphatidylinositol bisphosphate (PIP_2_), generating the active form phosphatidylinositol trisphosphate (PIP_3_). PIP_3_ binds to serine-threonine kinase Akt (protein kinase B) and causes its translocation to the membrane where it contacts phosphoinositoside-dependent kinase-1 (PDK1), which in turn leads to the phosphorylation and activation of Akt [142]. 

Akt is a key signaling kinase which contributes to DNL via the following mechanisms. (1) Akt phosphorylation and activation of the mammalian target of rapamycin complex (mTORC1) [145,146,147] supports lipogenesis through both SREBP-dependent and -independent mechanisms. mTORC1 phosphorylates and inactivates lipin-1 [148], which is a negative regulator of nuclear SREBP1c, leading to the sequestration of lipin-1 in the cytoplasm and increased SREBP-transcriptional activity [147,148]. mTORC1 also can regulate DNL independently of SREBPs through sequential phosphorylation and activation of ribosomal protein S6 kinase β-1 (S6K1) and the splicing factor SRSF protein kinase 2 (SRPK2), thereby increasing mRNA splicing of genes involved in DNL including ACLY, FASN and ACSS2 [149]. (2) Akt stabilizes mature SREBP1c through inhibition of GSK3β, which phosphorylates and promotes ubiquitination and proteasomal degradation [150]. (3) Akt directly activates ACLY to generate acetyl-CoA [151], and phosphorylates NADK to produce NADP^+^ for NADPH synthesis [152]. NADPH sustains the anabolic reaction of acetyl-CoA and malonyl-CoA catalyzed by FASN [152]. PI3K signaling also induces mTORC2 activation to support DNL through AKT-dependent and -independent mechanisms. In the AKT-independent pathway, mTORC2 phosphorylates serum- and glucocorticoid-induced protein kinase 1 (SGK1) and protein kinase C (PKC), subsequently activating SREBP1c [153,154].

In addition to the PI3K/Akt/mTORC pathway, the p53 and Hippo tumor suppressor pathways cross-talk with the SREBP network to stimulate mevalonate pathway genes and sterol biosynthesis [155,156,157]. Large tumor suppressor kinase 2 (LATS2) is a central regulator in Hippo pathway which regulates cell proliferation and differentiation [158]. LATS2 binds to the ER-tethered precursor of SREBP and inhibits processing and maturation [159]. Inactivation of LATS2 occurs in many cancers resulting in SREBP activation and cholesterol accumulation [158,159]. LATS2 and p53 tumor suppressors positively regulate each other. Wild-type p53 binds and transactivates the LATS2 promoter while LATS2 activates and stabilize p53 [160]. 

The p53 tumor suppressor is frequently mutated in many human cancer genomes [161]. In OvCa, a considerably high mutation frequency of 50–100% in *TP53* gene has been reported across all ovarian cancers [44,49,50,51,52]. This variation in *TP53* mutation frequency is due to the different histopathological subtypes of OvCa. Mutations in *TP53* gene are less frequent in low-grade serous or borderline tumors, but are ubiquitous in high-grade serous tumors [162,163]. *TP53* mutations result in three different effects that contribute to tumorigenesis: a loss-of-function (LoF) effect that deprives its tumor suppressor function, a dominant-negative (DN) effect in which the mutant allele masks the function of the wild-type allele, and a gain-of-function (GoF) effect which acquires novel oncogenic capabilities [51]. Wild-type p53 transcriptionally represses the expression of SREBP1 and its lipogenic target genes FASN and ACLY [164]. In addition, through transcriptionally inducing the ATP-binding cassette transporter (ABCA1) gene, wild-type p53 also inhibits the maturation of SREBP2 to reduce the expression of mevalonate pathway genes such as HMGCR, MVK, MVD etc., [165]. In contrast, cancer-derived missense GoF mutation forms of p53 are associated with SREBP and function as a transcriptional coactivator to upregulate enzymes in mevalonate pathway [155,156,166]. p53 null mutation or ablation of ABCA1 is associated with increased SREBP2 maturation and activation of the mevalonate pathway to promote liver tumorigenesis [165]. Together these findings reveal the integrated and coordinated regulation of SREBP-directed metabolic homeostasis by multiple oncogenic and tumor-suppressor signaling networks. 

### 4.4. SREBP1 and Ovarian Cancer

The presence of large volumes of malignant ascitic fluid provides OvCa with a unique tumor microenvironment [54,64,65,66,67]. Lysophosphatidic acid (LPA), a growth factor-like lipid mediator, is highly elevated in the ascites of OvCa patients and malignant effusions [167,168,169]. Host cells and tissues in the OvCa TME, such as adipocytes and mesothelial cells in the omentum and peritoneum constitutively produce LPA, which acts as a potent chemotactic mediator for OvCa metastasis [169,170,171]. In vitro studies demonstrated that LPA functions through LPA_2_, an LPA receptor subtype overexpressed in OvCa, to activate SREBP1 in OvCa cells [33,35]. SREBP1 stimulates de novo lipogenesis through upregulation of lipogenic enzymes such as FASN, ACC and HMGCR [33]. LPA regulation of SREBP1 can also stimulate glycolysis through induction of hexokinase 2 to promote OvCa cell proliferation [35]. Compared with the benign and the borderline human ovarian tumors, immunohistochemical staining showed that SREBP1 expression was significantly higher in the invasive subtypes of OvCa [34]. In a xenograft SCID mouse model, shRNA-mediated knockdown of SREBP1 inhibited ovarian tumor growth in vivo and reduce the expression of downstream target genes of SREBP1 [34]. 

Recently, we developed a highly integrative approach with three distinct preclinical animal models to examine the impact of host obesity on OvCa metastatic success [28]. Our results clearly demonstrated that obesity led to significantly enhanced overall and organ-specific metastatic burden in three in vivo cohorts: (1) nude mice fed a control diet (CD) vs. western diet (WD, 40% fat, diet-induced obesity, or DIO) injected with human OvCa cells; (2) C57Bl/6 mice fed CD vs. WD and injected with syngeneic murine ID8 OvCa cells; and (3) wild-type C57Bl/6 mice vs. B6.Cg-Lep^ob^ (leptin mutant, *ob/ob* mice) injected with ID8 cells. Examination of mechanisms by which obesity enhances metastatic success identified striking upregulation of SREBP1 in tumors from mice fed a WD and from *ob/ob* mice. SREBP1 showed intense nuclear localization, indicative of transcriptional activity that contributes to lipogenic reprogramming of tumor cells [28]. Furthermore, enhanced expression of FABP4 was observed in WD and *ob/ob* mice relative to controls, with intense staining present in adipocytes and in tumor cells immediately adjacent to adipocytes. Taken together, these data demonstrate that SREBP1-directed upregulation of de novo lipogenesis, lipid transport and uptake, as well as glycolysis contributes to enhanced OvCa growth and metastasis. 

## 5. Targeting SREBP1-Mediated Lipid Metabolism Pathways in Cancer Treatment

### 5.1. Targeting SREBP1 with Small Molecule Inhibitors

The enhanced reliance on lipids observed in models of cancers and obesity suggests the potential for new therapeutic targets and agents. SREBP1 functions as a central regulator of DNL and contributes to enhanced cancer growth and metastasis, particularly under obese conditions. Inhibition of SREBPs genetically or pharmacologically suppresses tumor growth significantly and induces cancer cell death, suggesting that SREBPs are emerging as promising therapeutic targets. Since transcription factors are usually considered as challenging direct targets [172,173], various ways to indirectly inhibit SREBPs were developed [38,103]. In recent preclinical trials, fatostatin, betulin, PF-429242 and BF-175 have been reported to inhibit SREBP activation and show antitumor effects in many cancers.

#### 5.1.1. Fatostatin

Fatostatin, a diarylthiazole derivative, directly binds to SCAP and blocks SREBP translocation from the ER to the Golgi thus inhibiting the processing and maturation of SREBP [174,175]. In obese mice, fatostatin treatment reduces adiposity and lowers hyperglycemia [174]. Studies have shown that fatostatin demonstrated antitumor effects, such as inhibition of cancer cell proliferation, invasion, and migration, and arresting cancer cells in G2/M phase in endometrial carcinoma [176], breast cancer [177], prostate cancer [178,179], pancreatic cancer [180], and colon cancer [132]. Fatostatin’s anticancer properties are attributed not only to its inhibition of SREBP-dependent processes on lipid metabolism, but also to its inhibition of microtubule formation and cell division [181].

#### 5.1.2. Betulin

Betulin is a pentacyclic triterpenoid which inhibits the ER-to-Golgi translocation of SREBPs through binding to SCAP in an INSIG-dependent manner [182]. Betulin inhibits DNL by suppressing the maturation of SREBP, therefore decreasing lipid content and diet-induced obesity [182]. The antiproliferative potential and cytotoxicity of betulin have been studied in a range of cancer cell lines and xenograft models, including liver cancer, prostate cancer, lung cancer, breast cancer and ovarian cancer [135,183,184,185,186,187]. In hepatocellular carcinoma, betulin suppress tumor progression by downregulating inflammatory cytokines, including IL-6, TNF alpha, and IL-1b [187]. Betulin also reduces glycolytic activity and synergizes sorafenib-mediated suppressive effect on hepatocellular carcinoma growth and metastasis [135]. 

#### 5.1.3. PF-429242

PF-429242 is a reversible amino-pyrrolidineamide inhibitor of site-1 protease (S1P), which inhibits endogenous SREBP processing and activation [188]. Inhibition of SREBP1 by PF-429242 led to decreased viability and proliferation in the pancreatic cancer cell line [180]. PF-429242 displayed synergistic anticancer activity with GSK343, a SAM-competitive inhibitor of the histone lysine methyltransferase EZH2 (enhancer of zeste homolog 2), in treating hepatocellular carcinoma [189]. In glioblastoma cells, PF-429242 inhibition of S1P and blocking SREBP reduced cholesterol levels, suppressed cell growth and induced apoptotic cell death [190].

#### 5.1.4. BF175

BF175, a boron-containing compound, can block the binding of SREBP1a transactivation domain to “mediator of RNA polymerase II transcription subunit 15-Kinase inducible domain interacting domain” (MED15-KIX). BF175 inhibits SREBP transcriptional activity, decreases lipogenic gene expression in cultured hepatocytes, and is effective in controlling diet-induced obesity in mice [191]. In aggressive lymphoma cell lines, “nuclear receptor subfamily 4, group A, member 3” (NR4A3) functions as tumor suppressor. BF175 treatment resulted in the induction of NR4A3-mediated apoptosis [100].

### 5.2. Targeting SREBP-Pathway Genes

In addition to pharmaceutical inhibition of SREBPs, targeting SREBP-regulated lipogenic enzymes has proven to be an effective treatment strategy against cancer metastasis, given the importance of lipogenesis in this process. Small molecule inhibitors of the key lipogenic enzymes, including FASN, ACLY, ACC, SCD, HMGCR, and CPT1, have been developed and are currently undergoing preclinical and clinical trials. 

#### 5.2.1. FASN

FASN is a key lipogenic enzyme catalyzing the last step in DNL of FAs. It catalyzes the synthesis of saturated FA palmitate from acetyl-CoA and malonyl-CoA in the presence of NADPH [86,192,193]. FASN expression is increased in various cancers [92,96,192,194]. Multiple FASN inhibitors, such as cerulenin [195,196], orlistat [197,198], C75 [199,200], C93 [201,202], GSK2194069 [203] and TVB-3166 [204] have demonstrated preclinical antitumor activity in variable cancer cell lines and xenograft models. 

New generation FASN inhibitors, including TVB-2640 [193] and omeprazole [205,206], have moved into the clinical studies currently (www.clinicaltrials.gov). TVB-2640 has entered three Phase 2 clinical trials in treating patients with lung cancer (NCT03808558) [207], breast cancer (NCT03179904) [208] and astrocytoma (NCT03032484) [209]. Two Phase 1 clinical trials studied the effects of TVB-2640 in colon cancer (NCT02980029) [210] and solid malignant tumor (NCT02223247) [211]. Omeprazole, an inhibitor of proton pump, effectively attenuates FASN to suppress DNL [205,206]. There are multiple clinical trials in Phase 2/3 stages evaluating the combinatory effects of omeprazole and standard chemotherapy in breast cancer (NCT02595372) [212], prostate cancer (NCT04337580) [213], and colorectal cancer (NCT02518373) [214].

#### 5.2.2. ACLY

ACLY is a SREBP-pathway downstream target that mediates the conversion of cytoplasmic citrate to acetyl-CoA. ETC-1002 (bempedoic acid), a dual ACLY inhibitor/AMPK activator, has effectively reduced the low-density lipoprotein and cholesterol in Phase 2/3 clinical trials in patients with hypercholesterolemia (NCT03001076, NCT01941836) [215,216], hyperlipidemia with cardiovascular disease (NCT02991118) [217] and type 2 diabetes (NCT01607294) [218]. Current clinical studies focus on inhibition of ACLY in treating dyslipidaemia. However, considerable preclinical evidence supports the integral role of ACLY in tumorigenesis and the potential as a target for anticancer drugs [121]. ACLY inhibitors such as SB-204990 suppress tumor growth in mice with lung, prostate or OvCa xenografts [90,97]. 

#### 5.2.3. ACC

Following the conversion of citrate and acetate to acetyl-CoA, ACC catalyzes ATP-dependent carboxylation of acetyl-CoA, generating malonyl-CoA for fatty acid biogenesis. ACC is upregulated in several human cancers including OvCa [98,137]. ACCs inhibitors TOFA, soraphen A and ND646 significantly reduce fatty acid synthesis and suppress tumor growth in various xenograft models [122,137,138,139,219,220]. 

#### 5.2.4. SCD

SCD catalyzes the desaturation of palmitate to form MUFAs such as oleic acid (18:1) or palmitoleic acid (16:1), which are substrates for membrane phospholipids, cholesteryl esters and triglycerides. Inhibition of SCD1 by SSI-4 [221], MF-438 [222,223], and betulinic acid [224] suppress tumor growth in preclinical xenograft models. 

#### 5.2.5. HMGCR

HMGCR catalyzes the conversion of HMG-CoA to mevalonic acid, which is further metabolized to farnesyl pyrophosphate, a precursor of cholesterol and sterols. Statins are HMGCR inhibitors that are widely used in the clinic to treat hypercholesterolemia. There have been multiple reports demonstrating that statins can exhibit anticancer activity preclinically and in patients [157,225,226,227]. However, studies have shown that statin-mediated inhibition of cholesterol synthesis can lead to feedback activation of SREBPs [228]. To make the anticancer properties of statins more effective, combination therapies that inhibit both cholesterol synthesis and SREBP activation simultaneously are in development [229,230].

#### 5.2.6. CPT1

CPT1 is the rate limiting enzyme that converts FAs to acylcarnitines, which are imported into mitochondria for β-oxidation [19,85,86]. CPT1expression is upregulated in many cancers. Energy produced from fatty acid oxidation (FAO) is critical in tumor growth [231,232]. Etomoxir is a specific CPT1 inhibitor that is highly effective at inhibiting FAO and has been evaluated for anticancer effects in various cancers. Etomoxir reduces ibrutinib resistance and inhibits leukemia cell proliferation [233,234]. Combinatorial treatments using etomoxir and glutaminase inhibitor CB-839 decreased growth and migration of CB-839-resistant triple negative breast cancer cells [235]. Etomoxir was found to synergize with orlistat, a drug that blocks lipid synthesis, to inhibit multiple myeloma [236] and prostate cancer cell proliferation and decrease xenograft growth [237]. Preclinical colorectal cancer [238] and OvCa [239] in vivo models found that targeting CPT1-mediated FAO activation with etomoxir could effectively promote anoikis and decrease metastasis to inhibit tumor progression.

Taken together, given the important role and the complex regulatory network of SREBP-mediated lipogenesis and metabolic reprogramming, SREBPs and SREBP-pathway genes are emerging as promising therapeutic targets in many cancers, particularly in the context of host obesity. SREBP inhibitors have beneficial effects either alone or in combination therapeutic approaches in treating cancers as shown in numerous preclinical animal models and human clinical trials. A better molecular understanding of the relationships between SREBP regulation and obesity in cancer progression might ultimately empower new therapeutic intervention strategies. Due to the involvement of SREBPs in many important metabolic pathways, potential cytotoxicity and off-target effects need to be carefully evaluated. Furthermore, considering the complex regulatory network and feedback loops, distinct components of the SREBP-pathway may need to be targeted simultaneously.

## 6. Conclusions

In summary, extensive studies strongly support the essential role of the SREBP1-regulated lipid metabolism network as a metabolic hub whose dysregulation can drive ovarian cancer progression and metastasis, particularly in the obese host. Altered expression of key SREBP1-pathway molecules involved in lipid uptake, lipid synthesis, desaturation, and fatty acid oxidation are linked to lipid-driven oncogenic progression and omental dissemination in OvCa. Current preclinical research and early phase clinical trials are focusing on lipid synthesis inhibitors which have shown promising anticancer effects. Pharmaceutical targeting of SREBPs, lipogenic enzymes, or lipid transporters in the SREBP1-pathway, either alone or in combination with standard chemotherapeutic drugs or other inhibitors represents an attractive therapeutic strategy. As their synergistic effects can help to eliminate OvCa cancer cells, including drug-resistant cells, future studies aimed at developing a deeper understanding of the mechanisms that regulate lipid synthesis, storage, utilization and efflux in OvCa cells are warranted.
